# Sprifermin (rhFGF18) modulates extracellular matrix turnover in cartilage explants ex vivo

**DOI:** 10.1186/s12967-017-1356-8

**Published:** 2017-12-12

**Authors:** Ditte Reker, Cecilie F. Kjelgaard-Petersen, Anne Sofie Siebuhr, Martin Michaelis, Anne Gigout, Morten A. Karsdal, Christoph Ladel, Anne C. Bay-Jensen

**Affiliations:** 1 Biomarkers and Research Rheumatology, Nordic Bioscience A/S, Herlev Hovedgade 205-207, 2730 Herlev, Denmark; 2 Osteoarthritis Research, Merck-KGaA, Frankfurter Strasse 250, 64293 Darmstadt, Germany; 3 Department of Biology, University of Copenhagen, Universitetsparken 13, 2100 Copenhagen Ø, Denmark; 4 Technical University of Denmark, Anker Engelunds Vej 1, 2800 Kgs. Lyngby, Denmark

**Keywords:** Osteoarthritis, Chondrocyte and cartilage biology, Other therapeutics

## Abstract

**Background:**

Sprifermin (recombinant human fibroblast growth factor 18) is in clinical development as a potential disease-modifying osteoarthritis drug (DMOAD). In vitro studies have shown that cartilage regenerative properties of sprifermin involve chondrocyte proliferation and extracellular matrix (ECM) production. To gain further insight into the process of sprifermin in the cartilage tissue, this study aimed at investigating the ECM turnover of articular cartilage explants in a longitudinal manner.

**Methods:**

Bovine full-depth articular cartilage explants were stimulated with sprifermin or placebo at weekly intervals, similar to the dosing regimen used in clinical trials. Pre-culturing with oncostatin M and tumour necrosis factor-α, was also used to induce an inflammatory state before treatment. Metabolic activity was measured using AlamarBlue, and chondrocyte proliferation was visualized by immuno-histochemical detection of proliferating cell nuclear antigen. ECM turnover was quantified by biomarker ELISAs; ProC2 reflecting type II collagen formation, CS846 reflecting aggrecan formation, active MMP9, C2M and AGNx2 reflecting matrix metalloproteinase activity, and AGNx1 reflecting aggrecanase activity.

**Results:**

Sprifermin was able to reach the chondrocytes through the extracellular matrix, as it increased cell proliferation and metabolic activity of explants. ProC2 and CS846 was dose-dependently increased (*P* < 0.05) by sprifermin compared to placebo, while C2M and AGNx2 were unaffected, active MMP9 was slightly decreased, and AGNx1 was slightly increased. Over the course of treatment, the temporal order of ECM turnover responses was AGNx1, then ProC2, followed by CS846 and MMP9. Pro-inflammatory activation of the explants diminished the ECM turnover responses otherwise observed under non-inflammatory conditions.

**Conclusions:**

The data suggest that sprifermin has chondrogenic effects on articular cartilage ex vivo, exerted through a sequential process of ECM turnover; aggrecan degradation seems to occur first, while type II collagen and aggrecan production increased at a later time point. In addition, it was observed that these chondrogenic effects are dependent on the inflammatory status of the cartilage prior to treatment.

**Electronic supplementary material:**

The online version of this article (10.1186/s12967-017-1356-8) contains supplementary material, which is available to authorized users.

## Background

Osteoarthritis (OA) is in urgent need of novel therapies that are not limited to alleviating symptoms, but rather have the ability to interfere with disease progression. As such, disease-modifying OA drugs (DMOADs) may target any of the joint tissues involved in OA, either by inhibiting the degenerative processes or by inducing regeneration. Sprifermin (recombinant human fibroblast growth factor 18, rhFGF18), is a novel DMOAD candidate that specifically targets articular cartilage and induces its regeneration.

The cartilage regenerative properties of sprifermin have been confirmed in vivo, by delivering sprifermin directly to the joint cavity as intraarticular injections once weekly. Animal studies have shown that intraarticular sprifermin prevent cartilage degradation and stimulate repair of damaged cartilage in traumatic OA models [1, 2], and has the ability to improve cartilage repair following micro-fracture treatment of chondral defects in an ovine model [3]. A first-in-man study in OA patients scheduled for knee joint replacement, delivered first indications that intraarticular sprifermin may have anabolic effects on joint cartilage by stimulating chondrocyte proliferation and positively influencing histological and biomechanical properties [4]. Later, in a phase Ib clinical study of intraarticular sprifermin, the primary end point, reduction of cartilage loss in the central medial femorotibial compartment, was not met. However, the secondary end points were consistent with a dose-related treatment effect on the cartilage across the total femorotibial joint and in the lateral femorotibial compartment [5]. Lastly, 2-year primary data from the currently ongoing 5-year phase II study was presented at this year’s American College of Rheumatology (ACR) Annual Meeting, showing a dose-dependent increase in total femorotibial joint cartilage thickness [6]. Thereby, the primary end point was met. No local or systemic serious safety concerns have been reported with intraarticular sprifermin [4–6].

In vitro studies have shown that sprifermin mainly activates the FGF receptor 3 (FGFR3) on the surface of the chondrocytes [7, 8]. By signalling through this receptor sprifermin induces cell proliferation [1, 2, 8–10] and formation of extracellular matrix (ECM) molecules; type II collagen [3, 7–10] and aggrecan [7–11], while promoting the chondrocyte phenotype [9, 10]. Together these results indicate that sprifermin induces cartilage formation by increasing the chondrocyte population and thereby allow increased ECM production. We have recently published data showing this effect in response to once weekly administration of sprifermin [10]. However, with permanent administration of sprifermin, cell proliferation was strongly stimulated at the expense of matrix production [10]. This indicates that the process of cartilage regeneration is a delicate balance between cellular and extracellular changes.

With the present study, we aimed at investigating the temporal changes in ECM turnover of articular cartilage explants in response to sprifermin, to gain insight into the longitudinal processes induced in the cartilage tissue. To do this, ex vivo articular cartilage explants were stimulated with sprifermin or placebo at weekly intervals to mimic the weekly intraarticular injections used in the clinic. The first aim was to confirm that anabolic effects previously observed in vitro [1–3, 7–11] could also be detected in explants, i.e. whether the chondrocytes could be reached through the matrix, and if chondrocytes in their physiological environment respond similarly than in cell culture. This was done by monitoring cell proliferation, metabolic activity and ECM formation (ProC2). Next, we speculated that chondrocyte proliferation may be accompanied by some degree of ECM degradation to expand the lacunae, and that ECM turnover (both formation and potential degradation) may be a function of time of treatment. Therefore, the second aim was to investigate the anabolic effects and potential catabolic effects over time, by monitoring ECM turnover biomarkers (ProC2, CS846, C2M, AGNx2, active MMP9 and AGNx1). Lastly, we speculated that the cartilage regenerative effects observed in vivo may be influenced by the inflammatory status of the joint cavity. Hence, the third aim was to investigate the influence of pro-inflammatory cytokines, oncostatin M (OSM) and tumor necrosis factor alpha (TNF-α), on sprifermin-induced ECM turnover (ProC2, C2M, AGNx2 and AGNx1).

## Methods

### Bovine full-depth articular cartilage explant culture

To investigate how both cellular and extracellular components of articular cartilage were affected by sprifermin compared to placebo, the well-described bovine full-depth articular cartilage explant model was used [12–18]. The effect parameters were tested on articular cartilage retrieved from three cows to account for biological variance.

#### Reagents

The culture medium was composed of DMEM/Nutrient Mixture F-12 with GlutaMAX (DMEM/F12-GlutaMAX) with 1% Penicillin Streptomycin (PS) both obtained from Life Technologies. Phosphate buffered saline (PBS), human OSM and human insulin-like growth factor-I (IGF-I) were obtained from Sigma-Aldrich, and human TNF-α were from R&D systems. The sprifermin used was drug product formulated for intra-articular administration obtained from Merck-Serono together with the corresponding placebo formulation. The placebo formulation contained 7 mM Na_2_HPO_4_, 1.0 mM KH_2_PO_4_, 2.7 mM KCl (pH 7.3).

#### Tissue preparation

Full-depth articular cartilage explants were harvested from the femoral condyle of the hind leg of cows aged ≤ 2 years, retrieved from the local slaughterhouse maximum 2 days after slaughtering (Harald Hansen). A biopsy punch with a 3 mm diameter (Miltex) was used to isolate full-depth cartilage plugs (i.e. explants) extending from the superficial layer to the subchondral bone. The isolated explants (10–20 mg, 2–3 mm in height) were randomly distributed in 96-well culture plates, washed three times in sterile PBS and cultured in DMEM/F12-GlutaMAX with 1% PS at 37 °C, 5% CO_2_.

#### Study design

Three studies were performed and reported independently using cartilage explants retrieved from three independent cows, one cow per study. In Study 1, the explants were cultured for 3 weeks with stimulation by sprifermin (900 ng/mL) or placebo in one media change per week (72 h duration). Twelve replicate explants were included for each condition and three of these were isolated for immuno-histochemical analysis after 1, 2 and 3 weeks. In Study 2 and 3, the explants were pre-cultured for 1 week either without stimulation or with continuous stimulation by the cytokines OSM (5 ng/mL) and TNF-α (10 ng/mL) (OSM + TNF-α). The pre-culturing period was followed by a compound-culturing period of 5 weeks with stimulation by the following compounds in one media change per week (48 h duration: sprifermin (1, 10, 50, 100 and 500 ng/mL) or placebo in Study 2 and sprifermin (11, 33, 100, 300 and 900 ng/mL) or placebo in Study 3. Six replicate explants, distributed diagonally in the culture plate, were included for each condition. Control groups receiving IGF-I (100 ng/mL) as positive control for cartilage formation (ProC2) were included in all studies. Generally, the conditioned media were collected three times weekly and stored at − 20 °C for further analyses. At the same time, freshly prepared medium was added either with (once a week) or without (remaining days) compounds.

### Metabolic activity and cell proliferation

#### AlamarBlue assay

AlamarBlue reagent (Life Technologies) was used as a non-toxic reagent for quantifying the metabolic activity of the explants at termination of each study and periodically during culturing. Briefly, conditioned media were collected and 200 μL 10% AlamarBlue in culture medium was added to each well with explants and four empty control wells. After 3 h. of incubation at 37 °C, 5% CO_2_, 160 μL of the oxidized liquid was transferred to black microtiter plates and the fluorescence was read at 540 nm excitation and 590 nm emission wavelengths. The remaining 40 μL was discarded and the explants were washed three times in sterile PBS before fresh culture medium was added. The readout was corrected for background by subtracting the average of the four controls from the result of each explant.

#### Immuno-histochemical detection of proliferating cell nuclear antigen (PCNA)

Immuno-histochemical detection of PCNA was used to visualize cell proliferation in formalin-fixed paraffin-embedded (FFPE) tissue slides of explants. Briefly, isolated explants were fixed in 4% formaldehyde (Merck Millipore) for 2 h at RT, embedded in paraffin and cut in 5 μm sections. Sections went trough de-paraffination, antigen retrieval and blocking, before PCNA was bound by anti-PCNA monoclonal mouse antibodies (Dako) and visualized by Envision+/HRP anti-mouse antibodies (Dako) and DAB+ substrate-chromogen solution (Dako). Sections were then dehydrated and mounted. Sections incubated with monoclonal mouse IgG2a antibodies (Dako) or PBS as substitute for anti-PCNA antibodies were included as negative controls for unspecific binding.

### Biochemical markers of ECM turnover

#### ProC2 ELISA

The ProC2 ELISA (Nordic Bioscience) was used to quantify type II collagen formation [13]. The ProC2 competitive ELISA detects the PIIBNP propeptide (QDVRQPG) released during trimming of newly synthesized type II collagen by N-propeptidases in the ECM. Briefly the assay was prepared as following, a streptavidin-coated plate was coated with Biotin-QDVRQPG. Samples were added together with supernatant from the clone NB443-3-2-1, producing monoclonal antibodies recognizing the PIIBNP propeptide (QDVRQPG). EnVision (Dako), containing peroxidase-conjugated anti-mouse antibodies, was used as a detector. 3,3′-5,5′-tetramethylbenzidine (TMB) was used as substrate and standard stop solution (0.1 M H_2_SO_4_) was added. The colorimetric reaction was measured at 450 nm with reference at 650 nm on a standard laboratory plate reader.

#### Cs846 ELISA

The CS648 ELISA (IBEX Pharmaceuticals Inc.) was used to quantify aggrecan formation. The CS846 competitive ELISA detects the CS846 epitope on chondroitin sulphate chains of large fetal-like aggrecan [19]. This form of aggrecan is thought to be synthesized in adult cartilage during cartilage repair [20, 21], but needs to be released from cartilage by MMP and/or aggrecan cleavage, in order to be detected. Briefly the assay was prepared as following, a goat anti-mouse antibody-coated plate was coated with mouse CS846 antibody. Samples were added together with Biotin-CS846. Peroxidase-labelled Streptavidin was used as a detector. TMB was used as substrate and standard stop solution was added. The colorimetric reaction was measured at 450 nm with reference at 650 nm on a standard laboratory plate reader.

#### C2M ELISA

The C2M ELISA (Nordic Bioscience) was used to quantify matrix metalloproteinase (MMP)-mediated type II collagen degradation [14]. The C2M competitive ELISA detects the C-terminal peptide (KPPGRDGAAG^1053^) generated by MMP cleavage. Briefly the assay was prepared as following, a streptavidin-coated plate was coated with Biotin-KPPGRDGAAG. Samples were added together with the peroxidase-labelled monoclonal antibody NBCH001, recognizing the C-terminal peptide (KPPGRDGAAG^1053^). TMB was used as substrate and standard stop solution was added. The colorimetric reaction was measured at 450 nm with reference at 650 nm on a standard laboratory plate reader.

#### AGNx1 ELISA

The AGNx1 ELISA (Nordic Bioscience) was used to quantify aggrecanase-mediated aggrecan degradation [15]. The AGNx1 competitive ELISA detects the C-terminal peptide (NITEGE^373^) generated by aggrecanase cleavage. This biomarker recognizes all cleavage fragments with an exposed NITEGE epitope, thus this includes the 32 mer (described by Lees et al. [22]), fragments with the G1 domain and even larger fragments with hyaluronic acid still linked to the G1 domain. Briefly the assay was prepared as following, a streptavidin-coated plate was coated with Biotin-NITEGE. Samples were added together with the monoclonal antibody 1H11, recognizing the C-terminal peptide (NITEGE^373^). A goat anti-mouse antibody was used as a peroxidase-labelled detector. TMB was used as substrate and standard stop solution was added. The colorimetric reaction was measured at 450 nm with reference at 650 nm on a standard laboratory plate reader.

#### AGNx2 ELISA

The AGNx2 ELISA (Nordic Bioscience) was used to quantify MMP-mediated aggrecan degradation [16, 17]. The AGNx2 ELISA detects the fragment ^342^FFGVG-G2 by combining two monoclonal antibodies in a sandwich ELISA. Briefly the assay was prepared as following, the monoclonal antibody AF-28, recognizing the N-terminal neo-epitope (^342^FFGVG) generated by MMP cleavage of the sequence DIPEN^341–342^FFGVG, was used as a biotinylated catcher. Samples were added, and then the monoclonal antibody F78, recognizing the G2 domains of aggrecan, was used as a peroxidase-labelled detector. TMB was used as substrate and standard stop solution was added. The colorimetric reaction was measured at 450 nm with reference at 650 nm on a standard laboratory plate reader.

#### Active MMP9 ELISA

The active MMP9 competitive ELISA (Nordic Bioscience) detects the N-terminal fragment (FQTFEGDLKW) of proteolytically activated MMP9 (unpublished). Briefly the assay was prepared as following, a streptavidin-coated plate was coated with Biotin-FQTFEGDLKW. Samples were added together with a peroxidase-labelled monoclonal antibody, recognizing the N-terminal fragment (FQTFEGDLKW). TMB was used as substrate and standard stop solution was added. The colorimetric reaction was measured at 450 nm with reference at 650 nm on a standard laboratory plate reader.

### Statistics

Baseline-correction was performed individually for each explant by dividing the value at each time point by the baseline value (week 0). Placebo-correction was performed individually for each explant by dividing the value at each time point by the corresponding mean value (n = 6) of the placebo-treated group. Incremental area under the curve (iAUC) was calculated individually for each explant using the trapezoidal rule. Values reported were mean and standard error of the mean (SEM) of six replicate explants (n = 6) originating from one cow. All explants in a single study originated from one cow. Statistical comparisons were only performed within each study and therefore between explants from the same cow. Unpaired t-test were used for comparison of two treatment groups and one-way ANOVA was used for multiple comparisons to the placebo group by assuming normal distribution. Statistical tests were performed in GraphPad Prism 6.0. Significance levels are indicated by asterisks; *P < 0.05, **P < 0.01, ***P < 0.001.

## Results

### Sprifermin increases chondrocyte proliferation, metabolic activity and ECM formation in cartilage explants

To evaluate the anabolic effect of sprifermin on both the cellular and extracellular component of cartilage, cell proliferation, metabolic activity and formation of type II collagen were monitored in parallel in cartilage explants after 0, 1, 2 and 3 weeks of compound-culturing (Study 1).

**Fig. 1:**
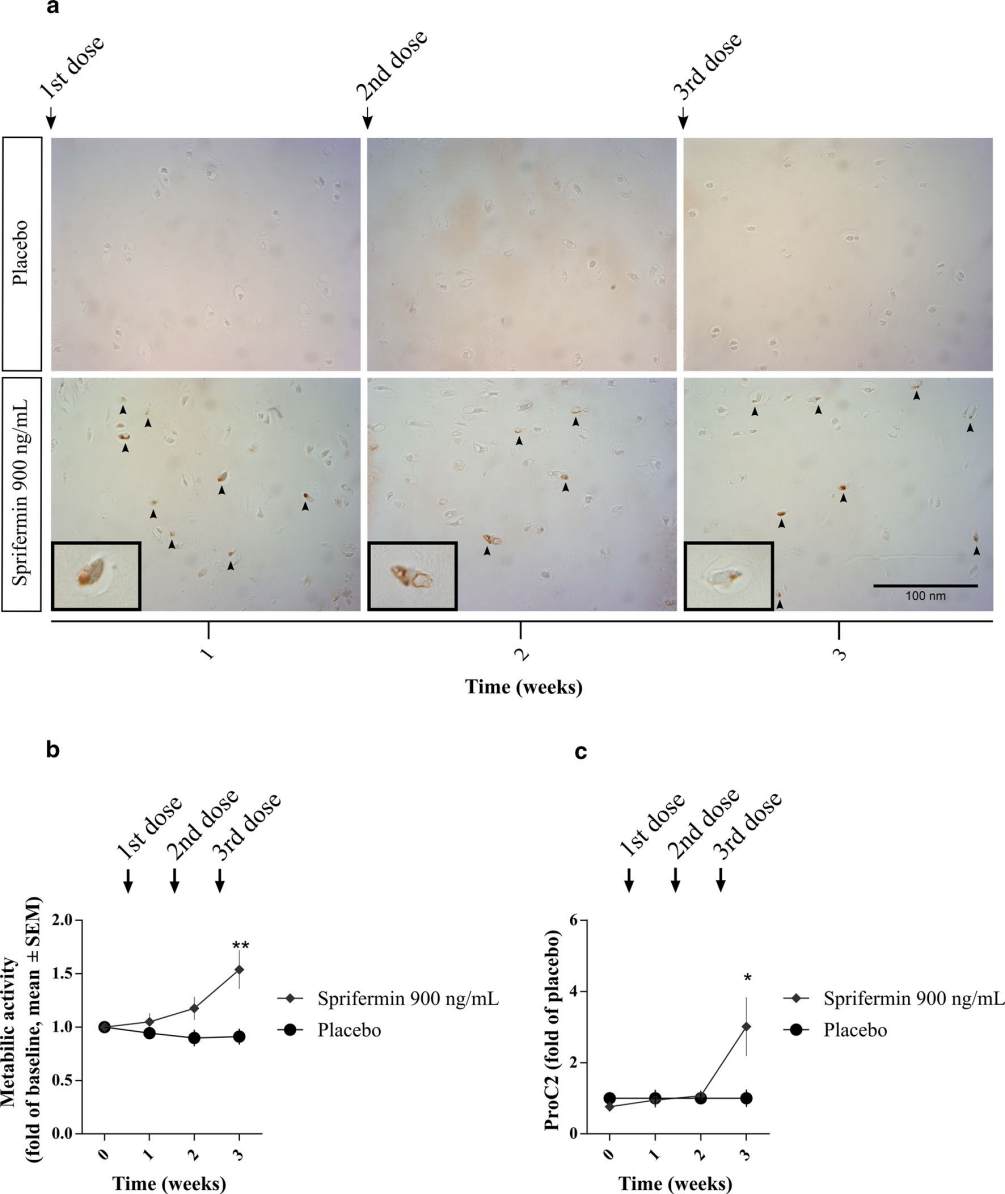
Cell proliferation, metabolic activity and type II collagen formation. Bovine cartilage explants were cultured for 3 weeks with weekly administration (72 h duration) of indicated compounds. **a** Cell proliferation was visualized by staining for PCNA in FFPE tissue slides from three replicate explants for each time point and treatment. One representative explant is shown. Scale bar applies to all pictures. **b** Metabolic activity was measured using AlamarBlue after 0, 1, 2 and 3 weeks of compound-culturing. Values were baseline-corrected and data presented as mean ± SEM of six replicate explants. **c** ProC2 (Nordic Bioscience) was measured in conditioned media collected at 0, 1, 2 and 3 weeks of compound-culturing. Values were placebo-corrected and data presented as mean ± SEM of six replicate explants. All results are from one study (Study 1). Unpaired t-test was used to compare the treatment group to the placebo group at each time point. Significance levels are indicated by asterisks; *P < 0.05, **P < 0.01, ***P < 0.001

Cell proliferation was detected by immuno-histochemical staining for PCNA, which is an essential protein for DNA-replication during cell proliferation. The presence of PCNA in chondrocytes was observed in explants isolated after 1, 2 and 3 weeks of culturing with sprifermin (900 ng/mL) stimulation once weekly, whereas almost none was observed in response to placebo (Fig. 1a). The same pattern was observed in 3 replicate explants for each time point and treatment. In parallel, metabolic activity of the same explants was continuously increased by sprifermin compared to placebo after 1, 2 and 3 weeks, when correcting for baseline-variance at day 0. After the third stimulation, the increase reached statistical significance (*P* = 0.009, Fig. 1b). To monitor the concurrent formation of type II collagen by the explants, the biomarker ProC2 was quantified in conditioned media harvested from these. IGF-I was the positive control of the ProC2 assay and IGF-I significantly increased ProC2 at all time points (P < 0.01) compared to placebo. ProC2 was significantly increased by sprifermin compared to placebo (*P* = 0.041, Fig. 1c), but only after the third stimulation. Hence, while cell proliferation and metabolic activity were affected already at the first stimulation by sprifermin, ProC2 release was observed at a later stage.

### Sprifermin modulates ECM turnover in cartilage explants over the course of treatment

To evaluate the temporal effect of sprifermin on ECM turnover in cartilage explants, the biomarkers ProC2, CS846, C2M, AGNx2, active MMP9 and AGNx1 were quantified in the conditioned media harvested from explants after 0, 1, 2, 3, 4 and 5 weeks of compound-culturing following pre-culturing in simple culture medium (Study 2 and 3).

**Fig. 2:**
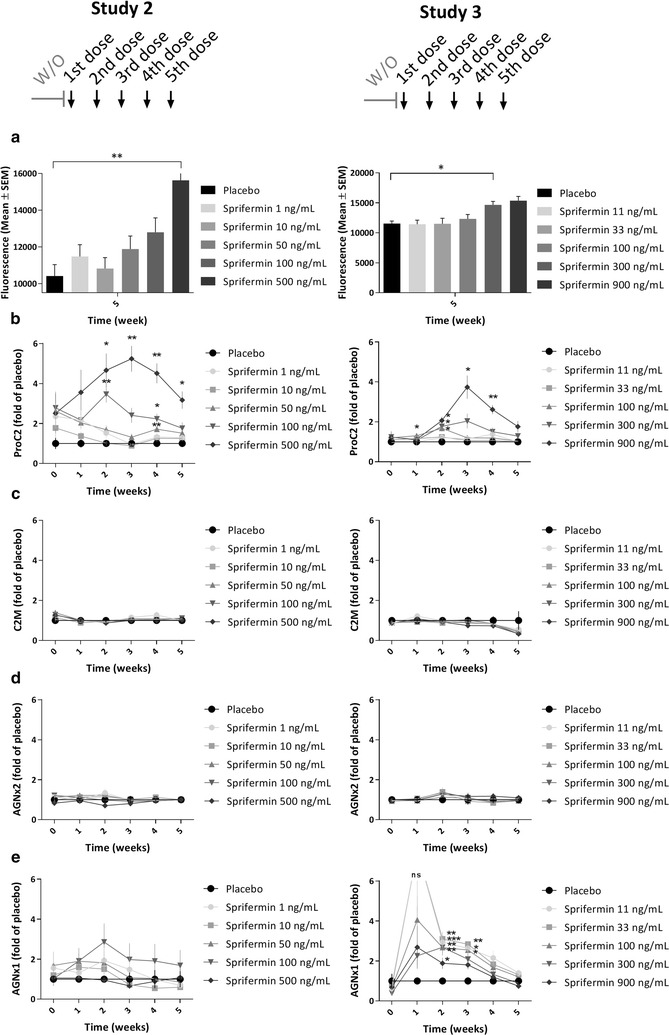
Metabolic activity and ECM turnover in bovine cartilage explants. Bovine cartilage explants were pre-cultured for 1 week, and then cultured for further 5 weeks with weekly administration (48 h duration) of indicated compounds. Metabolic activity (**a**) was measured using AlamarBlue after 5 weeks of compound-culturing. ProC2 (**b**), C2M (**c**), AGNx2 (**d**) and AGNx1 (**e**) (Nordic Bioscience) was measured in conditioned media collected at 0, 1, 2, 3, 4 and 5 weeks of compound-culturing. Values of biomarkers were placebo-corrected and all data presented as mean ± SEM of six replicate explants. One-way ANOVA was used for multiple comparisons to the placebo group at each time point. Significance levels are indicated by asterisks; *P < 0.05, **P < 0.01, ***P < 0.001. Results from two studies are shown in separate graphs (Left, Study 2; Right, Study 3)

First, the metabolic activity, quantified at termination of each study, was within the normal range for all explants, indicating sustained viability throughout the entire culturing period (Fig. 2a). Sprifermin dose-dependently increased metabolic activity compared to placebo, reaching statistical significance in both studies (500 ng/mL, *P* = 0.002; 300 ng/mL, *P* = 0.012).

ProC2, reflecting formation of type II collagen, was dose-dependently increased by sprifermin compared to placebo (Fig. 2b). The response reached statistical significance for the two highest doses at several time points (500 ng/mL, *P* ≤ 0.022; 900 ng/mL, *P* ≤ 0.022) compared to the placebo control. The peak effect of the highest dose in study 2 and 3 was observed after the third stimulation by sprifermin, being 5.2-fold higher than placebo in Study 2 and 3.7-fold higher in Study 3, when comparing mean values. The aggrecan formation biomarker, CS846, was dose-dependently increased by sprifermin compared to placebo (only evaluated in study 2) (Additional file 1). The peak effect of the highest dose was observed after the fifth stimulation, being 5.3-fold higher than placebo (*P* = 0.0002).

The catabolic biomarkers C2M and AGNx2, reflecting MMP-mediated degradation of type II collagen and aggrecan, respectively, were not affected by any of the investigated doses of sprifermin (Fig. 2c, d). Moreover, active MMP9 was slightly decreased by sprifermin compared to placebo, reaching a maximum of 0.6-fold decrease after the fifth stimulation by sprifermin (only evaluated in study 2) (Additional file 2). In contrast, the biomarker AGNx1, reflecting aggrecanase-mediated degradation of aggrecan, was increased by sprifermin compared to placebo (Fig. 2e). The peak effect was observed after the first or second stimulation by sprifermin, hence at an earlier stage than the ProC2 release described above. After the second sprifermin stimulation, the mean value of the 100 ng/mL dose was 2.9-fold higher than placebo mean value in Study 2 and 2.7-fold higher (*P* = 0.001) in Study 3.

These placebo-corrected comparisons at each specific time point (weeks 1–5) are not corrected for the variation at baseline (week 0), but the sprifermin-induction clearly exceeds baseline-variation. To address the baseline-variation, a baseline-corrected dataset, comparing the baseline level with the following weeks for each treatment group, is presented in additional material (Additional file 3).

### Pro-inflammatory pre-culturing changes the pattern of sprifermin-induced ECM turnover

To evaluate whether the inflammatory status of the cartilage explants affected the sprifermin-induced temporal changes of ECM turnover, cartilage explants were pre-cultured with OSM and TNF-α for 1 week prior to compound-culturing in parallel with the explants described above (only study 2). The biomarkers ProC2, C2M, AGNx2 and AGNx1 were quantified in the conditioned media harvested from the explants after 0, 1, 2, 3, 4 and 5 weeks of compound-culturing. Due to limitations in sample volume, AGNx2 could not be quantified at week 0.

**Fig. 3:**
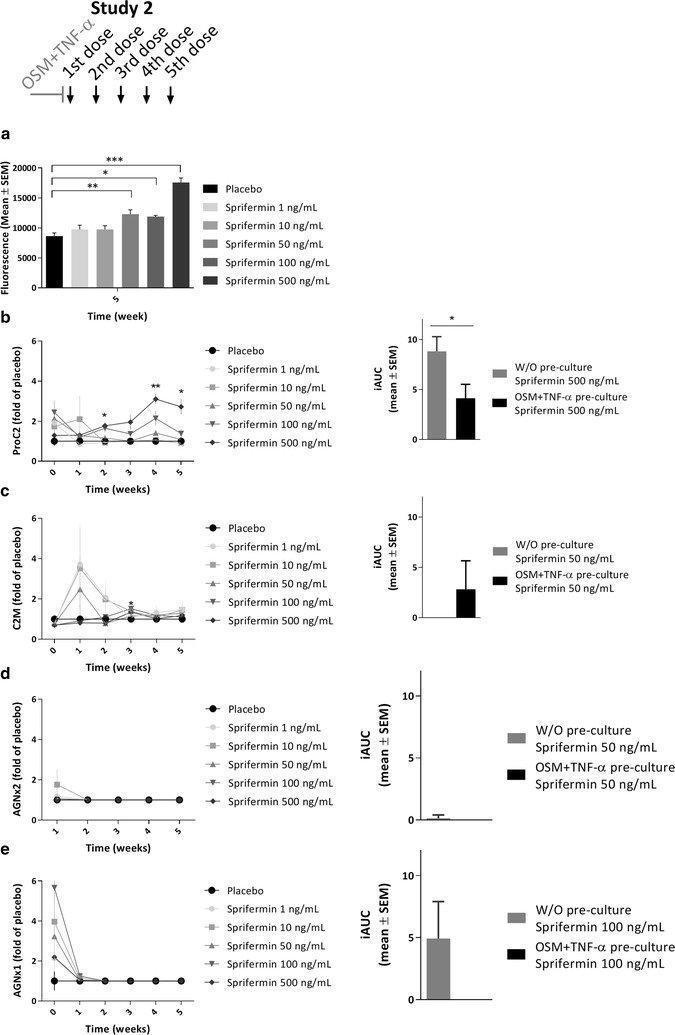
Metabolic activity and ECM turnover in pro-inflammatory bovine cartilage explants. Bovine cartilage explants were pre-cultured for 1 week with OSM + TNF-α (5/10 ng/mL), and then cultured for further 5 weeks with weekly administration (48 h duration) of indicated compounds. Metabolic activity (**a**) was measured using AlamarBlue after 5 weeks of compound-culturing. ProC2 (**b**), C2M (**c**), AGNx2 (**d**) and AGNx1 (**e**) (Nordic Bioscience) was measured in conditioned media collected at 0, 1, 2, 3, 4 and 5 weeks of compound-culturing, and values were placebo-corrected. All values are mean ± SEM of six replicate explants. One-way ANOVA was used for multiple comparisons to the placebo group at each time point. To compare the biomarker responses following pro-inflammatory and non-inflammatory (Fig. 2) pre-culturing, the iAUC of similar doses under each condition were calculated, and presented as mean ± SEM of six replicate explants (graph to the right). Unpaired t-tests were used to compare the two conditions. Significance levels are indicated by asterisks; *P < 0.05, **P < 0.01, ***P < 0.001. All results are from one study (Study 2)

First, the metabolic activity, quantified at termination of the study, was within the normal range for all explants, indicating sustained viability throughout the entire culturing period (Fig. 3a). As seen with non-inflammatory pre-culturing, sprifermin dose-dependently increased metabolic activity compared to placebo, reaching statistical significance for the three highest doses (500 ng/mL, *P* = 0.0003; 100 ng/mL, *P* = 0.013, 50 ng/mL, *P* = 0.004).

Generally, the pro-inflammatory pre-culturing diminished the patterns of ECM turnover that were observed with once weekly stimulation by sprifermin. ProC2 showed a delayed response after the fourth stimulation by sprifermin (500 ng/mL) in OSM + TNF-α pre-cultured explants (*P* = 0.002), the mean value being 3.1-fold higher than the placebo mean value (Fig. 3b, left). The impact of OSM + TNF-α pre-culturing on ProC2 release was confirmed by directly comparing the iAUC of the highest dose for the two pre-culturing conditions, showing a statistically significant difference (*P* = 0.043) (Fig. 3b, right). C2M showed an inverse dose-dependent increase after the first and second stimulation by sprifermin in OSM + TNF-α pre-cultured explants, the maximum mean value of sprifermin (50 ng/mL) being 2.5-fold higher than the placebo mean value (Fig. 3c, left). By comparing the iAUC of sprifermin (50 ng/mL) for the two pre-culturing conditions, C2M release was slightly higher following OSM + TNF-α pre-culturing, though not statistically significant (Fig. 3c, right). AGNx2 was not affected by any of the investigated doses of sprifermin (Fig. 3d, left), which was also evident from the iAUC of sprifermin (50 ng/mL) for the two pre-culturing conditions (Fig. 3c, right). Lastly, sprifermin did not increase AGNx1 in OSM + TNF-α pre-cultured explants (Fig. 3e, left), as it did in explants pre-cultured in simple culture medium (Fig. 2e, left). This difference in response between the two pre-culturing conditions is evident from the iAUC of sprifermin (100 ng/mL) (Fig. 3e, right).

## Discussion

The data of the present study confirm anabolic effects of sprifermin previously observed in vitro [1–3, 7–11], i.e. cell proliferation, metabolic activity and ECM formation, but here demonstrated in ex vivo explant cultures. This indicates that sprifermin is able to (1) diffuse through the ECM to reach the chondrocytes and (2) activate a cellular response from chondrocytes in their physiological environment. To summarize, articular cartilage explants stimulated with sprifermin at weekly intervals had increased cell proliferation, increased metabolic activity, and increased formation of type II collagen and aggrecan. The last three may be due to increased activity of each cell, or simply a result of the expanding cell population, or a combination of both.

We think that the observed anabolic effects in the present study are likely to be similar to the events occurring in sprifermin-treated OA patients. The dose levels used are physiologically relevant and the dose regimen is similar to the one used in the clinic. In the phase II clinical trial, OA patients received two or four cycles of treatment 6 or 12 months apart, each consisting of three once weekly intraarticular injections of 30–100 μg sprifermin or placebo, and the results so far show a dose-dependent increase in total femorotibial joint cartilage thickness [6]. Together these clinical results and our ex vivo results indicate that sprifermin, when delivered directly to the joint cavity by intraarticular injections, induces an increase in cartilage volume, which is due to first chondrocyte proliferation and later type II collagen and aggrecan formation.

**Fig. 4:**
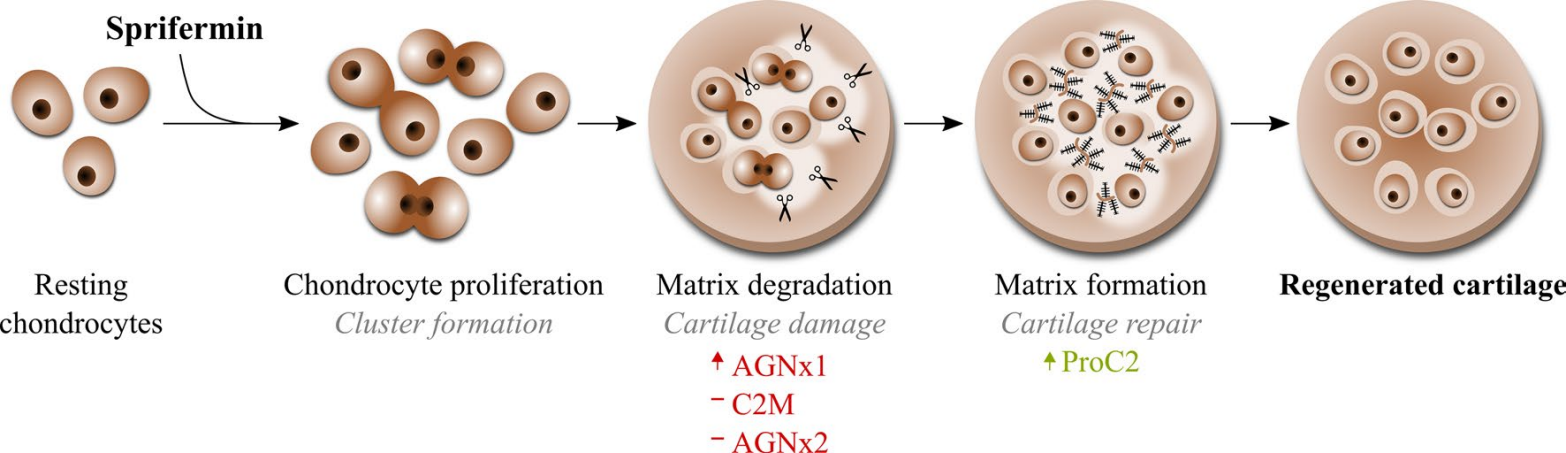
Sequential process of cartilage regeneration. Cartilage regeneration illustrated as a coordinated sequential process of chondrocyte proliferation and matrix turnover. The process may be initiated by sprifermin signaling to the chondrocytes to start proliferating. However, the chondrocytes are caged by the surrounding matrix, which preclude unlimited expansion. This may force the chondrocytes to initiate matrix degradation processes to expand the lacunae (get rid of their cage), and eventually we may see a larger population of chondrocytes that starts producing new matrix. Note that this figure illustrates a working hypothesis (and is hypothetical) as we do not know whether matrix degradation is required for regeneration of cartilage by pro-proliferative compounds like sprifermin

When evaluating the catabolic effects of sprifermin in this study, an increase of aggrecanase-mediated aggrecan degradation (AGNx1) was found, while MMP-mediated degradation was unchanged. Because sprifermin has been demonstrated to be an anabolic factor in several models in vitro or in vivo, this finding was unexpected. However, the lack of MMP-mediated aggrecan degradation may be attributed to the method used, and it could further be hypothesized that this aggrecanase-mediated aggrecan degradation occurs to enable chondrocyte proliferation. From ex vivo studies of cartilage degradation it is known that aggrecanases and MMPs act independently in the processing of aggrecan molecules. During cartilage degradation aggrecanase-mediated aggrecan degradation occurs in the initial phase, whereas MMP-mediated aggrecan degradation occurs at a later stage [15, 16]. Furthermore, the aggrecan loss occurring in the initial phase is completely reversible by IGF-I, while the aggrecan loss occurring at a later stage is irreversible [18]. Therefore, the aggrecanase-mediated aggrecan degradation should not be considered simply as an end-stage degradation process. When aggrecanase-mediated aggrecan degradation appears in context of a cartilage anabolic process, it may even be speculated that aggrecan degradation is part of a coordinated sequential process eventually leading towards cartilage regeneration. As illustrated in Fig. 4, such a cartilage regenerative process may hypothetically proceed as follows: resting chondrocytes residing in the cartilage ECM are stimulated by a pro-proliferative compound, like sprifermin, and start proliferating. However, expansion of the chondrocyte population is limited by the rigid ECM surrounding the chondrocytes. Thus, chondrocytes induce matrix degradation to expand the lacunae and allow proliferation, which is followed by ECM production, now by a greater chondrocyte population (Fig. 4). Although additional research is needed to confirm this observation, the slight increase in AGNx1 might suggest that matrix degradation is needed for expansion of lacunae, which may be needed to initiate the process of cell proliferation and cartilage formation.

Another aspect of AGNx1 release during cartilage regeneration is the potential bioactivity of the aggrecan fragment. A 32-mer fragment of aggrecan was shown to have both anti-anabolic, pro-catabolic and pro-inflammatory effects on chondrocytes, synovial fibroblast and macrophages in vitro [22]. This fragment contains the same C-terminal sequence (NITEGE^373^) as detected in the AGNx1 ELISA, but whether NITEGE alone has bioactive properties remains unknown. However, what is already known about the 32-mer fragment, clearly demonstrates that tissue remodelling may generate bioactive fragments, which do not only affect the tissue of origin, but may be released to the joint cavity and affect other joint tissues as well.

Interestingly, the results suggest that sprifermin is less potent in inducing an anabolic response in articular cartilage following inflammatory conditions as compared to non-inflammatory conditions. When explants were cultured with pro-inflammatory cytokines (OSM + TNF-α) prior to sprifermin treatment, little to no induction of type II collagen formation was observed, and the pattern of ECM degradation biomarkers was changed. Other studies have further shown that continuous co-culturing with OSM + TNF-α throughout the compound-culturing period completely abolished sprifermin-induced type II collagen formation (unpublished data). In a clinical context, this indicates that the cartilage regenerative effects of sprifermin may be influenced by the inflammatory status of the joint cavity. Hence, sprifermin may be a more effective DMOAD for OA patients with a low degree of synovitis, i.e. patients with expected lower levels of pro-inflammatory cytokines. Generally, such considerations of matching the right treatment with the right patient are highly important for succeeding in the development of novel OA therapies.

The present study was performed using full-depth articular cartilage explants to allow investigation of the cartilage tissue in its entirety. This model is limited to investigation of cartilage and do not account for the influence of other joint tissues or mechanical factors. The cartilage was of young bovine origin, hence is obviously more healthy than the cartilage of human OA patients, which is ultimately the target tissue of sprifermin. On the contrary, the human OA cartilage available would be from end-stage patients and may therefore already be too destroyed. Moreover, the large degree of patient-to-patient variation makes it difficult to get a statistically valid output from human OA cartilage.

## Conclusions

Sprifermin had an anabolic effect on articular cartilage, inducing cell proliferation, increased metabolic activity and formation of type II collagen. These chondrogenic effects seem to be accompanied by a sequential process of ECM turnover, initiated by aggrecanase-mediated aggrecan degradation, then type II collagen formation and eventually aggrecan formation. No indication of increased MMP activity was observed. After pro-inflammatory pre-activation of the cartilage, sprifermin was unable to induce formation of type II collagen to the same extent as after non-inflammatory pre-culturing, and the general pattern of ECM turnover was changed.

## Additional files

**Additional file 1.** Aggrecan formation in bovine cartilage explants. Bovine cartilage explants were pre-cultured for 1 week, and then cultured for further 5 weeks with weekly administration (48 h duration) of indicated compounds. CS846 (IBEX Pharmaceuticals Inc.) was measured in conditioned media collected at 1, 3 and 5 weeks of compound-culturing. Values were placebo-corrected and all data presented as means ± SEM of six replicate explants. One-way ANOVA was used for multiple comparisons to the placebo group at each time point. Significance levels are indicated by asterisks; *P < 0.05, **P < 0.01, ***P < 0.001. All results are from one study (Study 2).

**Additional file 2.** Active MMP9 in bovine cartilage explants. Bovine cartilage explants were pre-cultured for 1 week, and then cultured for further 5 weeks with weekly administration (48 h duration) of indicated compounds. Active MMP9 (Nordic Bioscience) was measured in conditioned media collected at 0, 1, 2, 3, 4 and 5 weeks of compound-culturing. Values were placebo-corrected and all data presented as means ± SEM of six replicate explants. One-way ANOVA was used for multiple comparisons to the placebo group at each time point. Significance levels are indicated by asterisks; *p < 0.05, **p < 0.01, ***p < 0.001. All results are from one study (Study 2).

**Additional file 3.** Baseline-corrected biomarker data. For each biomarker graph, the corresponding baseline-corrected dataset is presented. Values were baseline-corrected and data presented as means ± SEM of n replicate explants (n=6 if nothing else indicated). For the Fig. 1c dataset, unpaired t-test was used to compare the treatment group to the placebo group at each time point. For the Figs. 2 and 3 datasets, one-way ANOVA was used for multiple comparisons to the placebo group at each time point. P values are presented in parentheses.

## Abbreviations

ACR: american college of rheumatology
DMOAD: disease-modifying osteoarthritis drug
ECM: extracellular matrix
ELISA: enzyme-linked immunosorbent assay
FFPE: formalin-fixed paraffin-embedded
FGF: fibroblast growth factor
FGFR3: fibroblast growth factor receptor 3
GAG: glycosaminoglycan
iAUC: incremental area under the curve
IGF-I: insulin-like growth factor-I
MMP: matrix metalloproteinase
OA: osteoarthritis
OSM: oncostatin M
PBS: phosphate buffered saline
PCNA: proliferating cell nuclear antigen
PS: penicillin streptomycin
SEM: standard error of the mean
TMB: 3,3′-5,5′-tetramethylbenzidine
TNFa: tumor necrosis factor alpha
