# Supplementary figures and images for: Sprifermin (rhFGF18) modulates extracellular matrix turnover in cartilage explants ex vivo
Source: J Transl Med. 2017 Dec 12;15:250. doi: 10.1186/s12967-017-1356-8 (PMC5727954; doi:10.1186/s12967-017-1356-8)

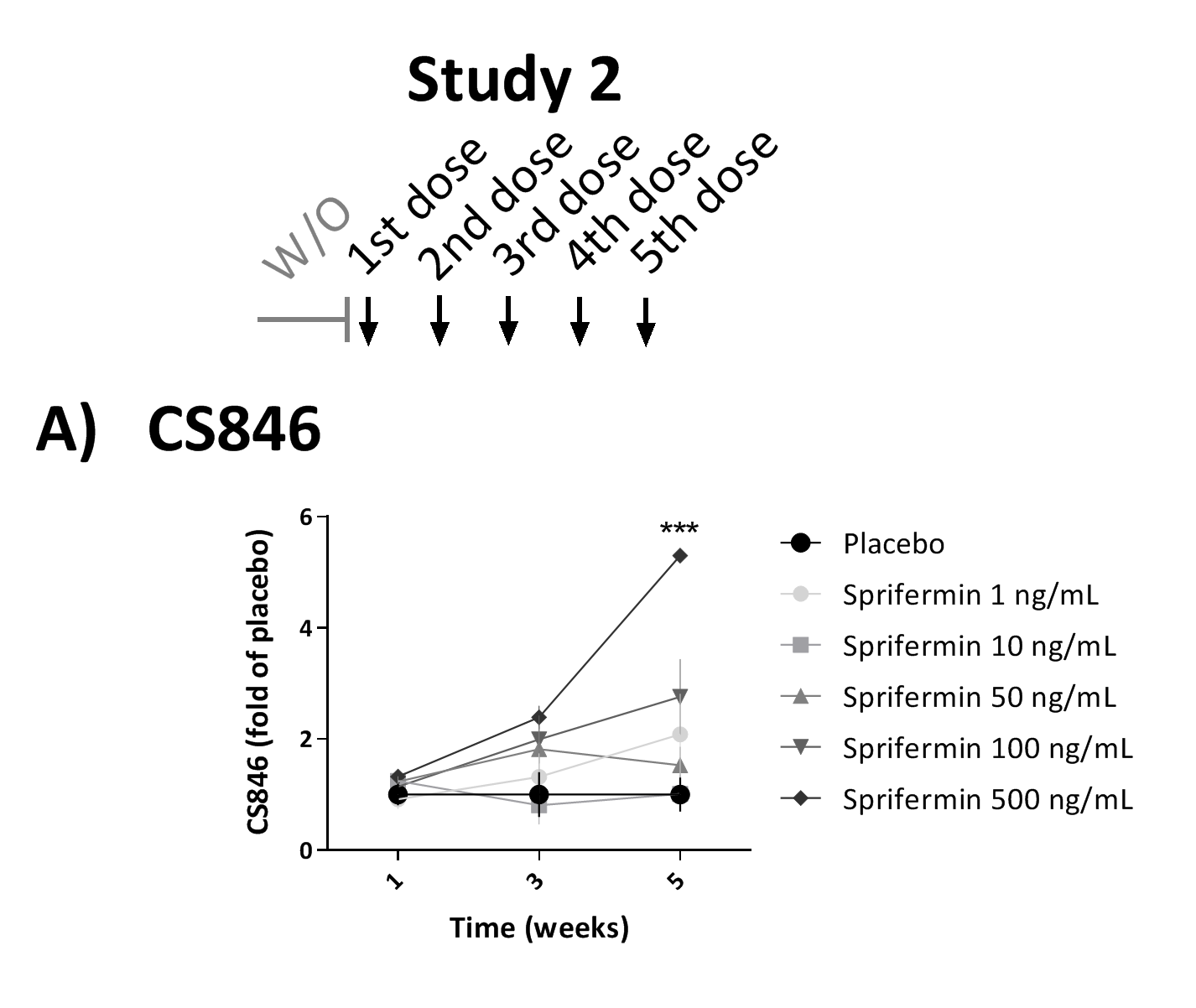

Supplement: Supplementary file 1 — Additional file 1. Aggrecan formation in bovine cartilage explants. Bovine cartilage explants were pre-cultured for 1 week, and then cultured for further 5 weeks with weekly administration (48 h duration) of indicated compounds. CS846 (IBEX Pharmaceuticals Inc.) was measured in conditioned media collected at 1, 3 and 5 weeks of compound-culturing. Values were placebo-corrected and all data presented as means ± SEM of six replicate explants. One-way ANOVA was used for multiple comparisons to the placebo group at each time point. Significance levels are indicated by asterisks; *P < 0.05, **P < 0.01, ***P < 0.001. All results are from one study (Study 2). [file 12967_2017_1356_MOESM1_ESM.tif]

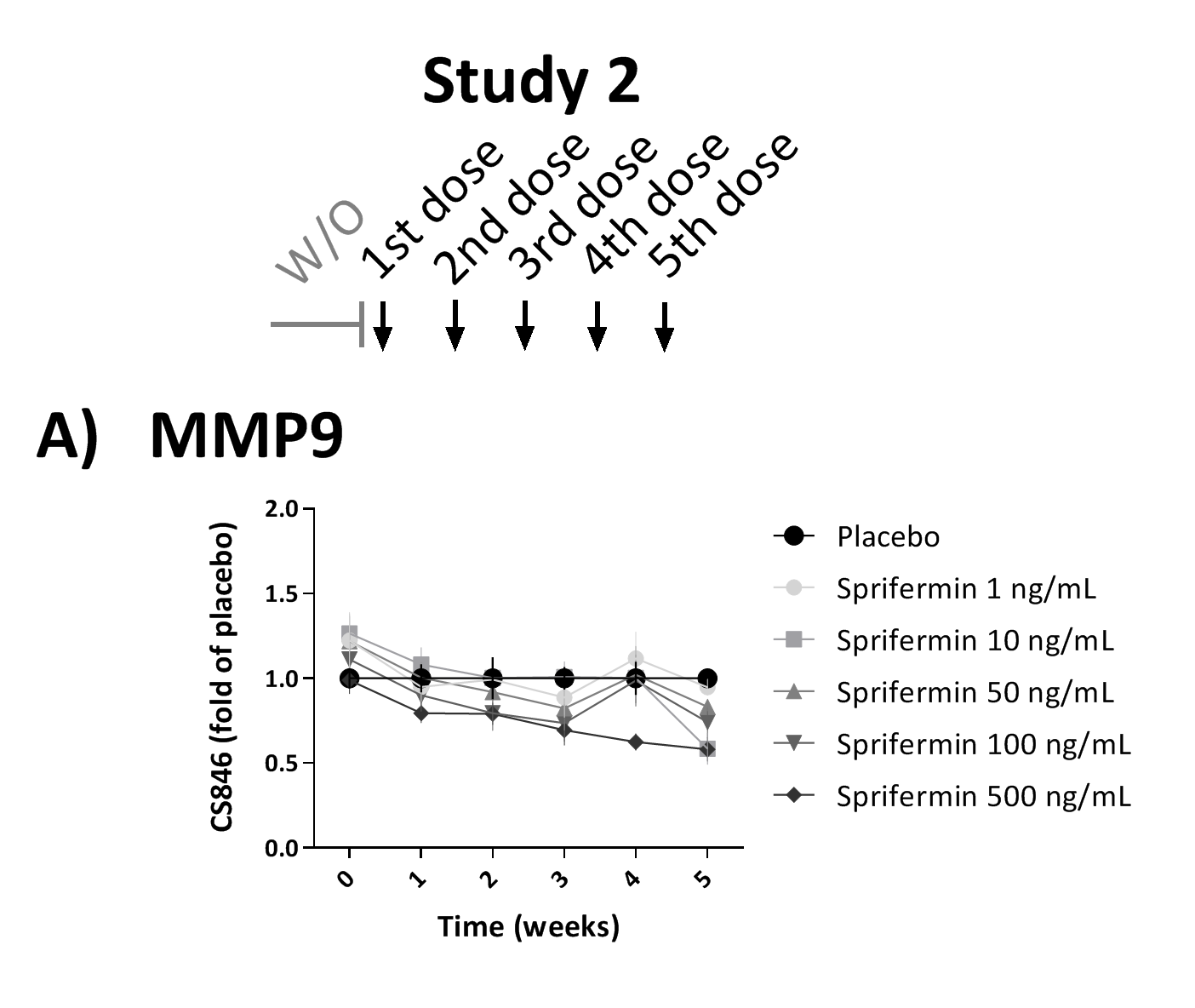

Supplement: Supplementary file 2 — Additional file 2. Active MMP9 in bovine cartilage explants. Bovine cartilage explants were pre-cultured for 1 week, and then cultured for further 5 weeks with weekly administration (48 h duration) of indicated compounds. Active MMP9 (Nordic Bioscience) was measured in conditioned media collected at 0, 1, 2, 3, 4 and 5 weeks of compound-culturing. Values were placebo-corrected and all data presented as means ± SEM of six replicate explants. One-way ANOVA was used for multiple comparisons to the placebo group at each time point. Significance levels are indicated by asterisks; *p < 0.05, **p < 0.01, ***p < 0.001. All results are from one study (Study 2). [file 12967_2017_1356_MOESM2_ESM.tif]
