# Supplementary material for: Sprifermin (rhFGF18) modulates extracellular matrix turnover in cartilage explants ex vivo
Source: J Transl Med. 2017 Dec 12;15:250. doi: 10.1186/s12967-017-1356-8 (PMC5727954; doi:10.1186/s12967-017-1356-8)
Supplement: Supplementary file 3 — Additional file 3. Baseline-corrected biomarker data. For each biomarker graph, the corresponding baseline-corrected dataset is presented. Values were baseline-corrected and data presented as means ± SEM of n replicate explants (n=6 if nothing else indicated). For the Fig. 1c dataset, unpaired t-test was used to compare the treatment group to the placebo group at each time point. For the Figs. 2 and 3 datasets, one-way ANOVA was used for multiple comparisons to the placebo group at each time point. P values are presented in parentheses. [file 12967_2017_1356_MOESM3_ESM.docx]

# ADDITIONAL MATERIAL

## Additional file 3: Baseline-corrected biomarker data.

## For each biomarker graph in the paper, the corresponding baseline-corrected dataset is presented. Values were baseline-corrected and data presented as means ± SEM of n replicate explants (n=6 if nothing else indicated). For the figure 1C dataset, unpaired t-test was used to compare the treatment group to the placebo group at each time point. For the figure 2 and 3 datasets, one-way ANOVA was used for multiple comparisons to the placebo group at each time point. P values are presented in parentheses.

| **Figure 1C. ProC2, Study 1** | | | | | | |
| --- | --- | --- | --- | --- | --- | --- |
|  | **Fold of baseline**  **(Mean ± SEM (P value, n≠6))** | | | | | |
| **Time (weeks)** | Placebo  (n=6) | Sprifermin 900 ng/mL  (n=6) |  |  |  |  |
| 0 | 1.000 ± 0.000  (-) | 1.000 ± 0.000  (-) |  |  |  |  |
| 1 | 0.341 ± 0.071  (-) | 0.415 ± 0.042  (0.3894) |  |  |  |  |
| 2 | 0.155 ± 0.021  (-) | 0.316 ± 0.127  (0.2412) |  |  |  |  |
| 3 | 0.069 ± 0.016  (-) | 0.403 ± 0.157  (0.0603) |  |  |  |  |
| **Figure 2B *left*. ProC2, Study 2** | | | | | | |
|  | **Fold of baseline**  **(Mean ± SEM (P value, n≠6))** | | | | | |
| **Time (weeks)** | Placebo  (n=6) | Sprifermin 500 ng/mL  (n=6) | Sprifermin 100 ng/mL  (n=6) | Sprifermin  50 ng/mL  (n=6) | Sprifermin  10 ng/mL  (n=6) | Sprifermin  1 ng/mL  (n=6) |
| 0 | 1.000 ± 0.000  (-) | 1.000 ± 0.000  (-) | 1.000 ± 0.000  (-) | 1.000 ± 0.000  (-) | 1.000 ± 0.000  (-) | 1.000 ± 0.000  (-) |
| 1 | 0.251 ± 0.042  (-) | 0.351± 0.038  (0.4938) | 0.233 ± 0.049  (0.9997) | 0.202 ± 0.031  (0.8977) | 0.184 ± 0.021  (0.7672) | 0.221 ± 0.040  (0.9946) |
| 2 | 0.253 ± 0.135  (-) | 0.363 ± 0.090  (0.9829) | 0.250 ± 0.061  (> 0.9999) | 0.126 ± 0.043  (0.8909) | 0.099 ± 0.020  (0.7789) | 0.110 ± 0.030  (0.8016) |
| 3 | 0.190 ± 0.058  (-) | 0.379 ± 0.068  (0.5189) | 0.165 ± 0.044  (0.9996) | 0.094 ± 0.034  (0.5908) | 0.080 ± 0.016  (0.5212) | 0.069 ± 0.017  (0.3145) |
| 4 | 0.181 ± 0.079  (-) | 0.286 ± 0.067  (0.9306) | 0.144 ± 0.056  (0.9979) | 0.094 ± 0.021  (0.8046) | 0.089 ± 0.015  (0.8115) | 0.073 ± 0.017  (0.6573) |
| 5 | 0.167 ± 0.059  (-) | 0.212 ± 0.065  (0.9957) | 0.112 ± 0.038  (0.9612) | 0.094 ± 0.032  (0.8462) | 0.096 ± 0.021  (0.7812) | 0.083 ± 0.026  (0.5300) |

| **Figure 2B *right*. ProC2, Study 3** | | | | | | |
| --- | --- | --- | --- | --- | --- | --- |
|  | **Fold of baseline**  **(Mean ± SEM (P value, n≠6))** | | | | | |
| **Time (weeks)** | Placebo  (n=6) | Sprifermin  900 ng/mL  (n=6) | Sprifermin  300 ng/mL  (n=6) | Sprifermin  100 ng/mL  (n=6) | Sprifermin  33 ng/mL  (n=6) | Sprifermin  11 ng/mL  (n=6) |
| 0 | 1.000 ± 0.000  (-) | 1.000 ± 0.000  (-) | 1.000 ± 0.000  (-) | 1.000 ± 0.000  (-) | 1.000 ± 0.000  (-) | 1.000 ± 0.000  (-) |
| 1 | 0.238 ± 0.024  (-) | 0.240 ± 0.035  (> 0.9999) | 0.239 ± 0.053  (> 0.9999) | 0.251 ± 0.021  (0.9735) | 0.251 ± 0.031  (0.9954) | 0.333 ± 0.098  (0.7762) |
| 2 | 0.100 ± 0.015  (-) | 0.169 ± 0.025  (0.0833) | 0.173 ± 0.046  (0.5061) | 0.127 ± 0.013  (0.6112) | 0.105 ± 0.016  (0.9983) | 0.170 ± 0.054  (0.6850) |
| 3 | 0.070 ± 0.012  (-) | 0.196 ± 0.024  (0.0046) | 0.123 ± 0.030  (0.4993) | 0.064 ± 0.009  (0.9777) | 0.061 ± 0.006  (0.8876) | 0.100 ± 0.026  (0.8543) |
| 4 | 0.073 ± 0.012  (-) | 0.152 ± 0.018  (0.0378) | 0.097 ± 0.022  (0.8143) | 0.066 ± 0.008  (0.8867) | 0.064 ± 0.007  (0.9032) | 0.130 ± 0.033  (0.5293) |
| 5 | 0.070 ± 0.012  (-) | 0.100 ± 0.022  (0.6881) | 0.088 ± 0.026  (0.9762) | 0.055 ± 0.007  (0.5932) | 0.058 ± 0.006  (0.8492) | 0.094 ± 0.027  (0.9382) |
| **Figure 2C *left*. C2M, Study 2** | | | | | | |
|  | **Fold of baseline**  **(Mean ± SEM (P value, n≠6))** | | | | | |
| **Time (weeks)** | Placebo  (n=6) | Sprifermin  500 ng/mL  (n=6) | Sprifermin  100 ng/mL  (n=6) | Sprifermin  50 ng/mL  (n=6) | Sprifermin  10 ng/mL  (n=6) | Sprifermin  1 ng/mL  (n=6) |
| 0 | 1.000 ± 0.000  (-) | 1.000 ± 0.000  (-) | 1.000 ± 0.000  (-) | 1.000 ± 0.000  (-) | 1.000 ± 0.000  (-) | 1.000 ± 0.000  (-) |
| 1 | 0.944 ± 0.056  (-) | 0.820 ± 0.153  (0.8639) | 0.753 ± 0.135  (0.6738) | 0.707 ± 0.100  (0.0400) | 0.758 ± 0.076  (0.1741) | 0.926 ± 0.103  (0.9998) |
| 2 | 0.964 ± 0.136  (-) | 0.675 ± 0.070  (0.5010) | 0.706 ± 0.070  (0.3623) | 0.675 ± 0.093  (0.4597) | 0.828 ± 0.128  (0.8758) | 0.844 ± 0.071  (0.9176) |
| 3 | 0.863 ± 0.067  (-) | 0.713 ± 0.107  (0.8227) | 0.685 ± 0.068  (0.5693) | 0.680 ± 0.070  (0.1213) | 0.810 ± 0.111  (0.9864) | 0.992 ± 0.160  (0.9717) |
| 4 | 0.835 ± 0.053  (-) | 0.675 ± 0.070  (0.4747) | 0.695 ± 0.073  (0.7144) | 0.656 ± 0.079  (0.4468) | 0.791 ± 0.086  (0.9936) | 1.047 ± 0.169  (0.7962) |
| 5 | 0.835 ± 0.053  (-) | 0.701 ± 0.095  (0.7769) | 0.723 ± 0.064  (0.8578) | 0.660 ± 0.076  (0.4601) | 0.747 ± 0.072  (0.7168) | 0.816 ± 0.066  (0.9997) |
| **Figure 2C *right*. C2M, Study 3** | | | | | | |
|  | **Fold of baseline**  **(Mean ± SEM (P value, n≠6))** | | | | | |
| **Time (weeks)** | Placebo  (n=6) | Sprifermin  900 ng/mL  (n=6) | Sprifermin  300 ng/mL  (n=6) | Sprifermin  100 ng/mL  (n=6) | Sprifermin  33 ng/mL  (n=6) | Sprifermin  11 ng/mL  (n=6) |
| 0 | 1.000 ± 0.000  (-) | 1.000 ± 0.000  (-) | 1.000 ± 0.000  (-) | 1.000 ± 0.000  (-) | 1.000 ± 0.000  (-) | 1.000 ± 0.000  (-) |
| 1 | 1.168 ± 0.166  (-) | 1.373 ± 0.191  (0.9271) | 1.238 ± 0.135  (0.9924) | 1.224 ± 0.267  (0.9998) | 1.299 ± 0.298  (0.9950) | 1.619 ± 0.254  (0.6544) |
| 2 | 1.014 ± 0.120  (-) | 1.008 ± 0.080  (> 0.9999) | 1.110 ± 0.247  (0.9941) | 1.143 ± 0.307  (0.9982) | 1.020 ± 0.199  (> 0.9999) | 1.174 ± 0.191  (0.9335) |
| 3 | 1.104 ± 0.205  (-) | 0.877 ± 0.090  (0.6310) | 1.105 ± 0.168  (> 0.9999) | 1.212 ± 0.162  (0.9147) | 1.228 ± 0.226  (0.9958) | 1.140 ± 0.207  (0.9999) |
| 4 | 1.016 ± 0.212  (-) | 0.790 ± 0.046  (0.7270) | 0.943 ± 0.159  (0.9996) | 0.930 ± 0.146  (0.9966) | 0.940 ± 0.157  (0.9996) | 0.928 ± 0.073  (0.9865) |
| 5 | 1.493 ± 0.275  (-, n=5) | 0.997 ± 0.243  (-) | 1.010 ± 0.192  (-) | 1.171 ± 0.125  (-) | 1.374 ± 0.151  (-) | 1.647 ± 0.154  (-) |
| **Figure 2D *left*. AGNx2, Study 2** | | | | | | |
|  | **Fold of baseline**  **(Mean ± SEM (P value, n≠6))** | | | | | |
| **Time (weeks)** | Placebo  (n=6) | Sprifermin  500 ng/mL  (n=6) | Sprifermin  100 ng/mL  (n=6) | Sprifermin  50 ng/mL  (n=6) | Sprifermin  10 ng/mL  (n=6) | Sprifermin  1 ng/mL  (n=6) |
| 0 | 1.000 ± 0.000  (-) | 1.000 ± 0.000  (-) | 1.000 ± 0.000  (-) | 1.000 ± 0.000  (-) | 1.000 ± 0.000  (-) | 1.000 ± 0.000  (-) |
| 1 | 0.764 ± 0.143  (-) | 0.893 ± 0.198  (0.9006) | 0.643 ± 0.077  (0.9598) | 0.738 ± 0.046  (0.9998) | 0.921 ± 0.192  (0.9902) | 0.616 ± 0.087  (0.9366) |
| 2 | 0.909 ± 0.167  (-) | 0.779 ± 0.169  (0.9615) | 0.661 ± 0.051  (0.6550) | 0.911 ± 0.129  (0.9999) | 1.061 ± 0.180  (0.9875) | 0.959 ± 0.142  (0.9976) |
| 3 | 0.615 ± 0.126  (-) | 0.614 ± 0.099  (0.9999) | 0.451 ± 0.073  (0.7069) | 0.505 ± 0.048  (0.8487) | 0.662 ± 0.117  (0.9997) | 0.482 ± 0.043  (0.8672) |
| 4 | 0.435 ± 0.090  (-, n=4) | 0.549 ± 0.103  (-, n=5) | 0.313 ± 0.027  (-) | 0.346 ± 0.016  (-) | 0.508 ± 0.106  (-) | 0.330 ± 0.016  (-, n=5) |
| 5 | 0.277 ± -  (-, n=1) | 0.602 ± 0.115  (-, n=4) | 0.305 ± 0.032  (-, n=5) | 0.326 ± 0.014  (-) | 0.501 ± 0.168  (-, n=4) | 0.303 ± 0.034  (-, n=2) |
| **Figure 2D *right*. AGNx2, Study 3** | | | | | | |
|  | **Fold of baseline**  **(Mean ± SEM (P value, n≠6))** | | | | | |
| **Time (weeks)** | Placebo  (n=6) | Sprifermin  900 ng/mL  (n=6) | Sprifermin  300 ng/mL  (n=6) | Sprifermin  100 ng/mL  (n=6) | Sprifermin  33 ng/mL  (n=6) | Sprifermin  11 ng/mL  (n=6) |
| 0 | 1.000 ± 0.000  (-) | 1.000 ± 0.000  (-) | 1.000 ± 0.000  (-) | 1.000 ± 0.000  (-) | 1.000 ± 0.000  (-) | 1.000 ± 0.000  (-) |
| 1 | 0.693 ± 0.114  (-) | 0.731 ± 0.061  (0.9996) | 0.730 ± 0.078  (0.9997) | 0.704 ± 0.100  (> 0.9999) | 0.746 ± 0.087  (0.9996) | 0.676 ± 0.077  (0.9999) |
| 2 | 0.277 ± 0.038  (-) | 0.386 ± 0.065  (0.3656) | 0.388 ± 0.047  (0.1423) | 0.340 ± 0.024  (0.2732) | 0.375 ± 0.067  (0.2868) | 0.369 ± 0.054  (0.0526) |
| 3 | 0.362 ± 0.085  (-) | 0.439 ± 0.060  (0.8222) | 0.403 ± 0.064  (0.9793) | 0.419 ± 0.078  (0.8789) | 0.340 ± 0.061  (0.9997) | 0.390 ± 0.055  (0.9953) |
| 4 | 0.176 ± 0.028  (-) | 0.221 ± 0.030  (0.4526) | 0.162 ± 0.015  (0.9269) | 0.163 ± 0.016  (0.9051) | 0.149 ± 0.012  (0.9232) | 0.191 ± 0.016  (0.9917) |
| 5 | 0.138 ± 0.007  (-) | 0.161 ± 0.014  (0.3358) | 0.135 ± 0.004  (0.9929) | 0.143 ± 0.009  (0.9980) | 0.141 ± 0.008  (0.9996) | 0.154 ± 0.022  (0.8050) |
| **Figure 2E *left*. AGNx1, Study 2** | | | | | | |
|  | **Fold of baseline**  **(Mean ± SEM (P value, n≠6))** | | | | | |
| **Time (weeks)** | Placebo  (n=6) | Sprifermin  500 ng/mL  (n=6) | Sprifermin  100 ng/mL  (n=6) | Sprifermin  50 ng/mL  (n=6) | Sprifermin  10 ng/mL  (n=6) | Sprifermin  1 ng/mL  (n=6) |
| 0 | 1.000 ± 0.000  (-) | 1.000 ± 0.000  (-) | 1.000 ± 0.000  (-) | 1.000 ± 0.000  (-) | 1.000 ± 0.000  (-) | 1.000 ± 0.000  (-) |
| 1 | 0.732 ± 0.122  (-) | 0.855 ± 0.130  (0.8101) | 1.547 ± 0.693  (0.7146) | 1.405 ± 0.544  (0.8277) | 0.939 ± 0.169  (0.9053) | 0.829 ± 0.123  (0.9956) |
| 2 | 0.859 ± 0.211  (-) | 0.836 ± 0.116  (0.9998) | 2.724 ± 1.138  (0.5327) | 1.598 ± 0.705  (0.9064) | 1.067 ± 0.266  (0.9904) | 2.788 ± 1.492  (0.7410) |
| 3 | 1.237 ± 0.569  (-) | 0.820 ± 0.131  (0.9322) | 2.644 ± 1.304  (0.8954) | 1.201 ± 0.453  (> 0.9999) | 0.681 ± 0.122  (0.8763) | 1.723 ± 0.710  (0.9946) |
| 4 | 1.553 ± 0.881  (-) | 1.428 ± 0.655  (0.9918) | 3.138 ± 1.631  (0.9422) | 1.483 ± 0.732  (> 0.9999) | 0.636 ± 0.125  (0.8315) | 1.440 ± 0.514  (0.9999) |
| 5 | 1.409 ± 0.738  (-) | 1.630 ± 0.855  (0.5773) | 2.549 ± 1.362  (0.9693) | 1.066 ± 0.414  (0.9983) | 0.636 ± 0.125  (0.8331) | 0.882 ± 0.175  (0.9583) |
| **Figure 2E *right*. AGNx1, Study 3** | | | | | | |
|  | **Fold of baseline**  **(Mean ± SEM (P value, n≠6))** | | | | | |
| **Time (weeks)** | Placebo  (n=6) | Sprifermin  900 ng/mL  (n=6) | Sprifermin  300 ng/mL  (n=6) | Sprifermin  100 ng/mL  (n=6) | Sprifermin  33 ng/mL  (n=6) | Sprifermin  11 ng/mL  (n=6) |
| 0 | 1.000 ± 0.000  (-) | 1.000 ± 0.000  (-) | 1.000 ± 0.000  (-) | 1.000 ± 0.000  (-) | 1.000 ± 0.000  (-) | 1.000 ± 0.000  (-) |
| 1 | 0.565 ± 0.190  (-) | 1.706 ± 0.937  (0.6714) | 1.622 ± 0.374  (0.1346) | 1.524 ± 0.276  (0.1056) | 3.892 ± 1.231  (0.1334) | 3.548 ± 1.331  (0.3133) |
| 2 | 2.175 ± 0.671  (-) | 6.242 ± 1.864  (0.4389) | 9.048 ± 1.958  (0.0994) | 6.991 ± 1.847  (0.0694) | 11.233 ± 2.575  (0.0719) | 9.244 ± 2.104  (0.0738) |
| 3 | 3.540 ± 1.495  (-) | 8.714 ± 2.426  (0.5881) | 10.460 ± 2.771  (0.3667) | 9.874 ± 2.921  (0.2369) | 15.482 ± 3.143  (0.0725) | 12.330 ± 3.197  (0.2216) |
| 4 | 4.719 ± 1.851  (-) | 7.525 ± 2.057  (0.9222) | 8.381 ± 2.172  (0.7661) | 8.120 ± 2.138  (0.2411) | 12.656 ± 2.902  (0.2365) | 13.561 ± 4.532  (0.3451) |
| 5 | 4.277 ± 1.722  (-) | 4.068 ± 1.187  (0.9999) | 4.915 ± 1.185  (0.9996) | 5.257 ± 1.397  (0.9530) | 8.459 ± 2.361  (0.6168) | 7.943 ± 2.316  (0.6129) |
| **Figure 3B *left*. ProC2, Study 2** | | | | | | |
|  | **Fold of baseline**  **(Mean ± SEM (P value, n≠6))** | | | | | |
| **Time (weeks)** | Placebo  (n=6) | Sprifermin  500 ng/mL  (n=6) | Sprifermin  100 ng/mL  (n=6) | Sprifermin  50 ng/mL  (n=6) | Sprifermin  10 ng/mL  (n=6) | Sprifermin  1 ng/mL  (n=6) |
| 0 | 1.000 ± 0.000  (-) | 1.000 ± 0.000  (-) | 1.000 ± 0.000  (-) | 1.000 ± 0.000  (-) | 1.000 ± 0.000  (-) | 1.000 ± 0.000  (-) |
| 1 | 1.432 ± 0.421  (-) | 1.514 ± 0.441  (0.9998) | 0.718 ± 0.218  (0.3301) | 0.727 ± 0.107  (0.3507) | 1.167 ± 0.501  (0.9982) | 2.920 ± 2.341  (0.9797) |
| 2 | 1.273 ± 0.287  (-) | 1.837 ± 0.503  (0.8371) | 0.885 ± 0.221  (0.7628) | 0.617 ± 0.109  (0.1638) | 0.558 ± 0.030  (0.2038) | 0.510 ± 0.080  (0.1121) |
| 3 | 0.662 ± 0.102  (-) | 1.101 ± 0.334  (0.6452) | 0.382 ± 0.063  (0.1436) | 0.299 ± 0.048  (0.0128) | 0.369 ± 0.058  (0.2675) | 0.309 ± 0.032  (0.0464) |
| 4 | 0.662 ± 0.102  (-) | 1.672 ± 0.405  (0.1656) | 0.584 ± 0.150  (0.9538) | 0.430 ± 0.101  (0.1285) | 0.383 ± 0.051  (0.2775) | 0.309 ± 0.032  (0.0464) |
| 5 | 0.719 ± 0.103  (-) | 1.746 ± 0.556  (0.3610) | 0.467 ± 0.168  (0.5267) | 0.366 ± 0.083  (0.0224) | 0.369 ± 0.058  (0.1780) | 0.309 ± 0.032  (0.0436) |
| **Figure 3C *left*. C2M, Study 2** | | | | | | |
|  | **Fold of baseline**  **(Mean ± SEM (P value, n≠6))** | | | | | |
| **Time (weeks)** | Placebo  (n=6) | Sprifermin  500 ng/mL  (n=6) | Sprifermin  100 ng/mL  (n=6) | Sprifermin  50 ng/mL  (n=6) | Sprifermin  10 ng/mL  (n=6) | Sprifermin  1 ng/mL  (n=6) |
| 0 | 1.000 ± 0.000  (-) | 1.000 ± 0.000  (-) | 1.000 ± 0.000  (-) | 1.000 ± 0.000  (-) | 1.000 ± 0.000  (-) | 1.000 ± 0.000  (-) |
| 1 | 2.069 ± 0.462  (-) | 2.121 ± 0.321  (0.9999) | 2.371 ± 0.229  (0.9918) | 6.270 ± 4.236  (0.8560) | 8.715 ± 5.228  (0.7323) | 9.126 ± 5.284  (0.6667) |
| 2 | 1.273 ± 0.334  (-) | 1.260 ± 0.131  (> 0.9999) | 1.731 ± 0.254  (0.8099) | 1.172 ± 0.177  (0.9996) | 2.724 ± 0.834  (0.5928) | 2.933 ± 1.217  (0.6955) |
| 3 | 1.575 ± 0.369  (-) | 2.588 ± 0.551  (0.1605) | 2.900 ± 0.330  (0.0502) | 2.120 ± 0.363  (0.8614) | 2.254 ± 0.400  (0.4612) | 1.776 ± 0.365  (0.9769) |
| 4 | 1.040 ± 0.130  (-) | 1.418 ± 0.074  (0.2795) | 1.648 ± 0.222  (0.2784) | 1.581 ± 0.218  (0.2829) | 1.292 ± 0.281  (0.9112) | 1.574 ± 0.330  (0.3850) |
| 5 | 1.217 ± 0.248  (-) | 1.814 ± 0.298  (0.3294) | 1.710 ± 0.250  (0.4364) | 1.826 ± 0.329  (0.6493) | 1.950 ± 0.201  (0.1625) | 1.944 ± 0.357  (0.3031) |
| **Figure 3D *left*. AGNx2, Study 2** | | | | | | |
|  | **Fold of baseline**  **(Mean ± SEM (P value, n≠6))** | | | | | |
| **Time (weeks)** | Placebo  (n=6) | Sprifermin  500 ng/mL  (n=6) | Sprifermin  100 ng/mL  (n=6) | Sprifermin  50 ng/mL  (n=6) | Sprifermin  10 ng/mL  (n=6) | Sprifermin  1 ng/mL  (n=6) |
| 0 | Not measured | Not measured | Not measured | Not measured | Not measured | Not measured |
| 1 | N/A | N/A | N/A | N/A | N/A | N/A |
| 2 | N/A | N/A | N/A | N/A | N/A | N/A |
| 3 | N/A | N/A | N/A | N/A | N/A | N/A |
| 4 | N/A | N/A | N/A | N/A | N/A | N/A |
| 5 | N/A | N/A | N/A | N/A | N/A | N/A |
| **Figure 3E *left*. AGNx1, Study 2** | | | | | | |
|  | **Fold of baseline**  **(Mean ± SEM (P value, n≠6))** | | | | | |
| **Time (weeks)** | Placebo  (n=6) | Sprifermin  500 ng/mL  (n=6) | Sprifermin  100 ng/mL  (n=6) | Sprifermin  50 ng/mL  (n=6) | Sprifermin  10 ng/mL  (n=6) | Sprifermin  1 ng/mL  (n=6) |
| 0 | 1.000 ± 0.000  (-) | 1.000 ± 0.000  (-) | 1.000 ± 0.000  (-) | 1.000 ± 0.000  (-) | 1.000 ± 0.000  (-) | 1.000 ± 0.000  (-) |
| 1 | 0.664 ± 0.178  (-) | 0.267 ± 0.062  (0.3970) | 0.205 ± 0.098  (0.4768) | 0.101 ± 0.018  (0.1130) | 0.104 ± 0.023  (0.0911) | 0.468 ± 0.182  (0.6834) |
| 2 | 0.664 ± 0.178  (-) | 0.266 ± 0.062  (0.3970) | 0.200 ± 0.100  (0.4734) | 0.100 ± 0.018  (0.1131) | 0.100 ± 0.025  (0.0921) | 0.468 ± 0.182  (0.6834) |
| 3 | 0.664 ± 0.178  (-) | 0.266 ± 0.062  (0.3970) | 0.200 ± 0.100  (0.4734) | 0.100 ± 0.018  (0.1131) | 0.100 ± 0.025  (0.0921) | 0.468 ± 0.182  (0.6834) |
| 4 | 0.664 ± 0.178  (-) | 0.266 ± 0.062  (0.3970) | 0.200 ± 0.100  (0.4734) | 0.100 ± 0.018  (0.1131) | 0.100 ± 0.025  (0.0921) | 0.468 ± 0.182  (0.6834) |
| 5 | 0.664 ± 0.178  (-) | 0.266 ± 0.062  (0.3970) | 0.200 ± 0.100  (0.4734) | 0.100 ± 0.018  (0.1131) | 0.100 ± 0.025  (0.0921) | 0.468 ± 0.182  (0.6834) |
